# Review on Advanced Cancer Modeling for a Cancer Study

**DOI:** 10.3390/cimb44110362

**Published:** 2022-10-31

**Authors:** Yong-Hee Cho

**Affiliations:** Data Convergence Drug Research Center, Therapeutics & Biotechnology Division, Korea Research Institute of Chemical Technology (KRICT), Daejeon 34114, Korea; y-hcho@krict.re.kr; Tel.: +82-42-860-7419

**Keywords:** organoid, cancer organoid, patient-derived tumor organoid, drug screening, patient-derived tumor xenograft

## Abstract

Intensive efforts to develop anti-cancer agents have been made for over 60 years. However, cancer is still considered a lethal disease. To study the best anti-cancer agents for improving the survival rates of cancer patients, many researchers have focused on establishing advanced experimental applications reflecting on the biomimetics of cancer patients involving the heterogeneity of cancer cells. The heterogeneity of cancer cells, which are derived from various clones and affected by different environments, presents different genetic backgrounds and molecular characteristics attributed to the differential responses to cancer therapies, and these are responsible for the resistance to cancer therapies, as well as for recurrence following cancer treatments. Therefore, the development of advanced applications for the cancer patient is expected to help the development of more effective anti-cancer agents. The present review evaluates recently developed cancer models encompassing the heterogeneity of cancer cells, which present similar morphological architecture, genetic backgrounds, and molecular characteristics to corresponding patient tumor tissues.

## 1. Introduction

Cancer is the leading cause of cancer-related deaths worldwide [1,2,3]. Although recent advances in the development of anti-cancer agents have been made in the field, the clinical benefits produced as a result of these drugs have not increased by a considerable amount because of the recurrences, resistances, and progressive nature of the disease [4,5,6]. Given that cancer cells consist of a heterogeneous population, which results in phenotypic variations, there is an increasing understanding that model systems containing heterogeneous cancer cells would be a breakthrough in the development of anti-cancer agents, improving the overall survival rate of cancer patients [7,8,9]. One of the challenges of developing effective anti-cancer agents is that the usage of cancer models recapitulates the patient’s tumor in the process of drug development to anticipate the response of new drugs to the clinical trials.

The inter- and intra-tumoral heterogeneities of cancer cells are among the most challenging aspects of tumor biology. Cancer cells are a complex and dynamic system where evolving cells both affect and are affected by the physiological properties of their environment [10]. Heterogeneity in genetic mutations, in gene expressions, and in protein modifications is considered as the major reason for driving the heterogeneity of cancer cells in the long process of tumorigenesis [11]. In relation to the heterogeneity of cancer cells, cancer stem cells (CSCs) are the main cause for generating the heterogeneity of cancer cells [12]. CSCs that are asymmetrically divided produce CSCs and non-CSCs as their differentiated progenitors [13]. They generate all cell types existing in tumors at the top of the tumor hierarchy [8,13]. A hierarchical organization of tumors governed by CSCs exists [8,9,13]. Recently, the origin of CSCs has been elucidated in the original population of colorectal cancer (CRC). The mutation of *apc* that occurs in LGR5^+^ intestinal stem cells allows them to be transformed into cancer stem cells, and LGR5^+^ CSCs play a critical role in the development of CRCs via producing the heterogeneity of tumors [14]. LGR5^+^ CSCs also play an essential role in the recurrence and metastasis of papillary cancer and CRC [5]. Although the characteristics of CSCs that possess initiation potential have been considered as main causes of the metastasis and recurrence of cancer, the molecular mechanisms have not yet been investigated and the exact phenomena have not been observed. Cho and Ro et al. (2020) elucidated and explained how CSCs are involved in the recurrence of CRCs. 

Recently, cancer models possessing the heterogeneity of cancer cells and recapitulating patient tumors have been developed and used as powerful experimental tools, such as an organoid system with high efficiency for cancer patients and patient-derived xenograft tumor (PDTX) [15,16,17,18]. They are becoming powerful applications for investigating personalized cancer treatments and drug development [19,20,21]. Recently developed 3D culture organoids derived from cancer cells are being expanded to the study of cancer and used by the medical society, allowing for more physiological human cancer studies in vitro [15]. The cancer cells with stem cell characteristics can grow into self-organizing spheroids, reflecting some structural aspects of native cancer tissues [22,23]. Recently, cancer organoids were successfully derived from induced pluripotent stem cells (iPSCs). However, the limited efficiency of successfully generated iPSCs-based cancer application is dependent on tumor type and their mutational status is a limitation at present [24,25]. 

Animal model systems recapitulating cancer have also been established. Patient-derived xenografts (PDTXs) have recapitulated their corresponding cancer tissues with similar genetic characteristics and histological architectures [19,26,27,28]. Since PDTXs consist of cancer cells, as well as of their neighboring stromal cells, the investigation of cancer mechanisms or the therapeutic effects of anti-cancer agents are available in the presence of the interconnection of cancer and stromal cells. Therefore, the PDTX model has been the main focus of the medical society and drug-development schemes. However, the high cost to establish and limit the usage of passages is a current limitation for high-throughput drug screening and personalized therapeutic strategies [17,19,26]. This review focuses on evaluating the characteristics of applications, to date, used for cancer research and personalized therapies.

## 2. Limitations of Conventional Cancer Cell Lines

**Figure 1:**
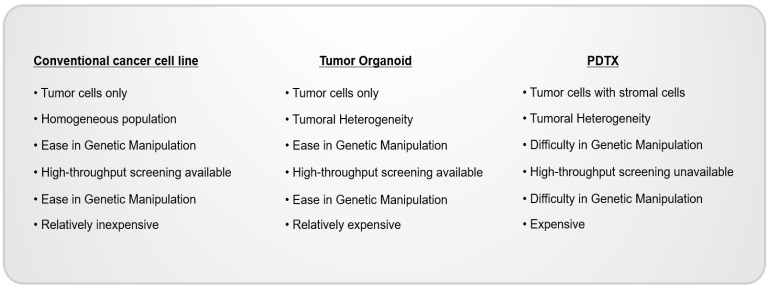
Overview of general cancer models, at present.

Given the relatively low cost, easy manipulation, and high-throughput availability of cancer cell lines, they have been used as the most general, conventional in vitro model in various disease studies and have especially contributed to the considerable achievements of cancer research [29]. However, accumulating evidence has revealed that tumors consist of various types of cells derived from different clones and are grown in different environments, which accelerates the diversity of cancer cells [30,31,32,33,34]. Indeed, recent, advanced, genomic analyses showed that every tumor comprises various clones, and even cancer cells derived from the same clone have different characteristics [35,36,37,38]. The homogeneous, conventional, cancer cell lines do not exhibit the diversity of cancer cells and do not represent the cancer patients’ tissues. The identification of the mechanisms underlying cancer development and the development of anti-cancer agents by using appropriate model systems containing the heterogeneity of cancer is essential. However, cancer cells derived from primary-patient cancer tissues have been used as advanced applications that contain the heterogeneity of cancer cells. However, their heterogeneity is not adequate enough, and passage usability is restricted. Recently, cancer model systems harboring the heterogeneity of cancer tissues, such as tumor organoids obtained from cancer patient cells and patient-derived tumor organoids (PDTXs), have been developed and used for cancer studies [21,39] (Figure 1).

Cancer model systems possess differential characteristics. The model systems can be used for various cancer studies, such as drug screening, mechanisms involved in cancer development in the presence of tumoral heterogeneity and the tumor microenvironment, and biomarker development for predicting drug responses. Determining the model systems suitable for each study helps to determine clinically and physiologically meaningful results. 

## 3. Cancer Organoid Model Systems 

Recently, cancer organoids retaining the similar genetic and phenotypic characteristics of their original cancer tissues and tumor subtype have been developed and considered as avatar model systems for cancer patients [40,41]. Cancer organoids have been used as a prominent application for preclinical and translational research [42]. The definition of organoids is a “mini-organ grown in vitro”. The organoids are self-organized, three-dimensional tissue cultures that are derived from normal, adult stem cells [22,23,43]. The adult stem cells divide indefinitely, producing all types of component cells as a part of their progeny. Histologically, organoids, three-dimensional culture-generating organs in vitro, were firstly developed by using mouse lgr5^+^ intestinal stem cells. Intestinal organoids, 3D-derived from lgr5^+^ stem cells, produce all kinds of differentiated cells comprising the intestine and form the structure of the intestine in vitro [43]. On the basis of recent reports that *apc* mutation occurring in lgr5^+^ stem cells is the origin of colorectal cancer (CRC), organoid model systems, using *apc*-mutated lgr5^+^ stem cells, have been used as a powerful CRC model system [14]. Moreover, living organoid biobanks have been developed by using the tumor cells of CRC patients. The cancer organoids closely recapitulate the heterogeneity of corresponding patient tumors, evaluated by the similarity of structure and genetic-mutation status, gene-expression analyses, and the sensitivity of organoids to anti-cancer agents compared with their original tumors. Given that the characteristics of tumor organoids derived from cancer patients faithfully recapitulate the architecture of their original tumor tissues and organoids, cancer-organoid-culture technology has expanded to other types of cancers. Many researchers have established long-term organoid cultures by using primary-colon [15,44], lung [45,46,47], esophageal [48], pancreatic [49,50,51], prostate [51,52,53], breast [30,54,55], stomach [56,57], liver [40,58], and endometrial [59,60] cancer and normal tissues. 

## 4. Applications for Investigating the Cancer Type Lacking Experimental Models

Not all diseases have model systems for investigating the diseases, such as the identification of biomarkers and developing therapeutic agents. Organoid model systems could be used as a powerful application for investigating diseases lacking experimental models [61,62]. Especially, given the scientific experiences for establishing protocols of various cancer types of organoids, organoid-culture systems have been considered as a potential model for investigating cancer types that lack experimental models [59,62]. Indeed, lung cancer, one of the most lethal types of cancer worldwide [63,64], is generally classified into non-small-cell lung cancer (NSCLC) and small-cell lung cancer (SCLC), comprising approximately 80% and 20% of all lung cancers, respectively [65,66]. Although distinct characteristics of SCLCs and NSCLCs exist, such as rapid doubling time and easy metastasis, few studies have investigated this area because of the lack of SCLC model systems [67,68]. Recently, Choi et al. (2020) established the organoids derived from SCLC with long-term expansions. These SCLC organoids reproduce the heterogeneity of cancer cells as shown by the recapitulation of original tumors, similar molecular expression patterns, and genomic characteristics [6]. Interestingly, the SCLC organoids generated in this study present a similar response to anti-cancer agents with corresponding cancer patients, suggesting that the SCLC organoids established in this study produce model systems, such as the clinical, usable avatar model system. 

## 5. Organoid Model Systems Produced by Using a Genetic Mouse Model 

Accumulating evidence has suggested that tumors consist of various types of cells, and their genetic diversity drives the process of tumorigenesis. Tumor organoids derived from cancer-patient tissues are confirmed as an application for patient avatar models [69]. Given the inter-patient, intra-tumoral, intra-clonal genetic diversity of cancer cells, an investigation of cancer mechanisms and drug development should be cautiously conducted to prevent the restricted usage of drug development or cancer mechanisms [69,70]. Tumor organoids using tumor cells obtained from genetic animal model systems have been used as an application for developing mechanisms involved in tumorigenesis and drug studies. Cho and Ro et al. (2020) presented the molecular mechanisms of organoids derived from the tumor cells of an *apc*-mutated mouse model. They identified the molecular mechanisms underlying 5-FU recurrence by using an *apc*-mutated mouse model, which occurs in over 90% of CRC patients [4,5]. These molecular mechanisms identified by using a general mouse model were beneficial for identifying that molecular mechanisms can be used for most cancer patients. 

## 6. The Limitations, at Present, of the Organoid Model System

Organoids derived from several cancer-type cells do not grow faster than those of matching normal cells [52,71], possibly because of their rates of mitotic failure, reduced telomerase activity, and oncogenic stress. Indeed, normal epithelial cell contamination presents a challenge to the use of organoids derived from non-small-cell lung cancer. The methods used to overcome tumor-purity problems have also been reported in the literature [72]. The determination of tumor purity is the first and most important step, and various methods have been suggested in the literature. Genetic analysis is the rapid and easy method used to determine the tumor-purity factor [73,74]. Copy number (CN) profiles were generated for 5 normal airway organoids (AOs) and 5 extra-pulmonary tumor organoids, and 20 intra-pulmonary tumor organoids with matching AOs. The tumor purity of lung cancer organoids was compared using intra- and extra-pulmonary tumors [72]. A systematic evaluation of the presence of well-known lung tumor-specific somatic mutations, such as EGFR, KRAS, FLT3, and STK11, was also reported. These methods are also useful to detect the presence overgrown, normal AOs. Histo-morphology and immuno-histo-chemistry (IHC) are powerful strategies used to determine the tumor purity level. Histo-morphological features by hematoxylin and eosin (H&E) analyses are methods used to easily classify the risk status of organoids. However, H&E analyses with an evaluation of IHC could aid in the development of a classifier. Thyroid transcription factor 1 (TTF-1), a marker normally expressed in type 2 pneumocytes and club cells in the lung and tumor protein 63 (P63), expressed in the basal cells of respiratory epithelium, is generally used [72]. 

**Figure 2:**
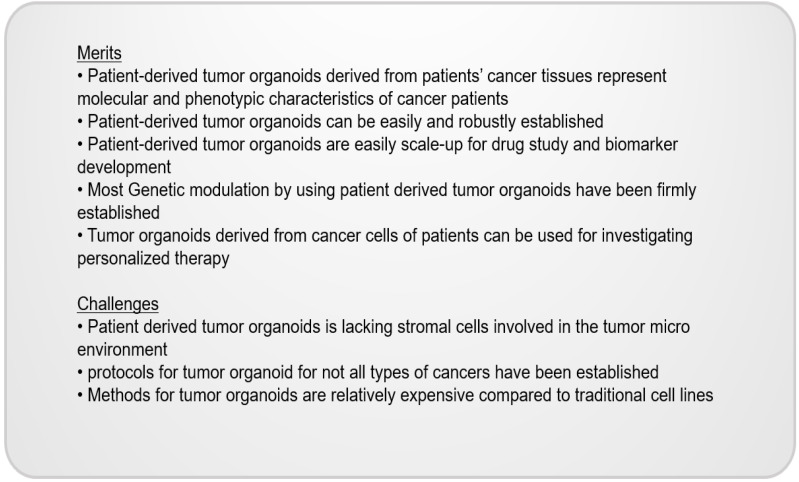
The benefits and limitations of organoid systems.

Recent studies have successfully established tumor organoids by using lung tissues, reducing growth factors and selecting tissue-specific growth factors. Kim et al. (2019) showed that reduced growth factors, such as EFG and FGF, successfully established the organoids derived from NSCLC and neighboring normal epithelial cells [7]. Choi et al. (2020) also demonstrated that reduced MAP kinase growth factors with the increase in Wnt agonists are essential or the long-term expansion of small-cell lung cancer (SCLC). Tumor organoids cultured by these methods are considered as a patient avatar model confirmed by genetic-similarity analyses, histological morphology differences, and molecular expression levels by the IHC of markers [6] (Figure 2). 

Merits and challenges exist in the current organoid system. Patient-derived tumor organoids are considered as patient avatar model systems representing the heterogeneity of patients’ cancer tissues. In addition, once established, the organoids are easily scaled up for high-throughput drug screening and large-scale genomic screening. However, in studies conducted by using tumor organoids, the effects of the tumor micro-environment (TME) on cancer cells are not involved since tumor organoids only consist of cancer cells without stromal cells. 

## 7. Patient-Derived Tumor Xenografts (PDTXs) as a Patient’s Avatar Model 

**Figure 3:**
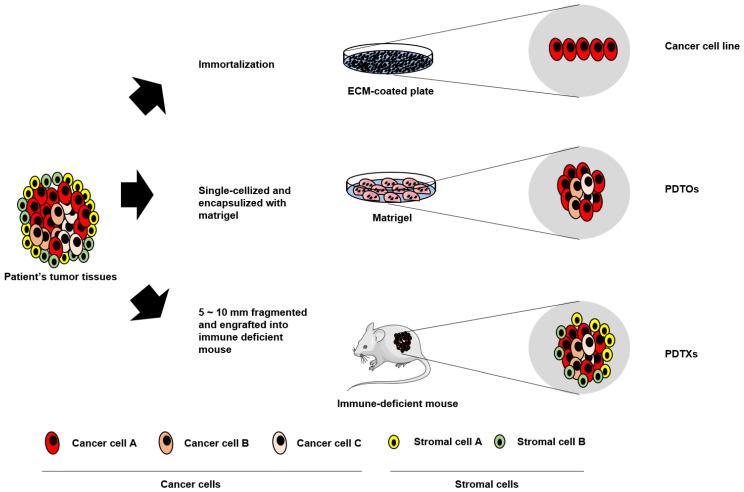
Establishment of cancer cell lines, PDTOs, and PDTXs.

Patient-derived tumor xenografts (PDTXs), transplanted tumor fragments surgically dissected from cancer patients and administered to immune-deficient mice, are an important model for translational and medical research [75]. PDTX model systems harbor the heterogeneity of cancer tissues and constitute tumors similar to original tissues with up to 14 passages. Given the differences in mouse and human conditions, PDTX model systems are emerging applications for replacing mouse tumor model systems [76,77]. Since a tumor fragment includes the patients’ stromal tissues, this model allows for translational research in the presence of tumor–stromal interactions [19,78]. However, PDTXs possess several issues. First, they have relatively low establishment rates and require a long period of time to be performed [19]. Second, establishing a PDX model system is costly and resource-intensive, limiting its statistical power. Therefore, PDTX model systems are not suitable for high-throughput drug screening [17,77]. To date, tumor organoids are the most effective application for drug screening (Figure 3). 

Conventional cancer cell lines, PDTOs, and PDTXs are established by different methods and have differential characteristics. Cancer cell lines are homogeneous and easily grown two-dimensionally in ECM-coated plates. PDTOs consist of heterogeneous populations of cancer cells and are three-dimensionally grown in scaffolders, including Matrigel. PDTXs, models engrafted on 5~10 mm fragmented cancer tissues into immune-deficient mice, reflect on TME, as well as tumoral heterogeneity.

ECM: extra-cellular matrix; PDTOs: patient-derived tumor organoids; PRTXs: patient-derived tumor xenografts; TME: tumor micro-environment.

## 8. Drug Study 

**Figure 4:**
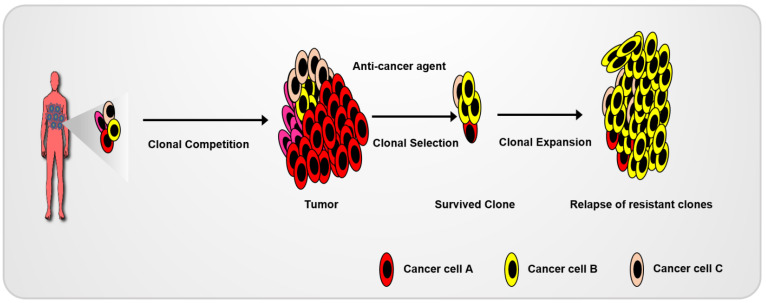
Diagram of recurrence in heterogeneous cancer cells.

The heterogeneity of cancer cells is responsible for the differential drug sensitivity and intrinsic resistance to anti-cancer agents (Figure 4). Recently, organoids have been successfully used for drug screening in the development of anti-cancer agents and testing their application in a clinical environment. Wetering et al. (2015) established living biobanks using colon-cancer-organoid-correlated molecular and genetic signatures. Using the living biobank, they developed a drug-screening platform by using a 3D-organoid culture [15]. Cancer organoids can act as a patient’s avatar model system for the use of high-throughput drug screenings to allow for the improvement of precision medicine. Given the mutational diversity of human CRC patient tumors, tumor organoids derived from mouse models harboring highly frequent mutations in certain cancers are also an effective method for developing cancer-mechanism studies. Cho and Ro et al. (2020) demonstrated the recurrence mechanisms following 5-FU-based therapy using organoids derived from general, genetic CRC animals and CRC patients. They identified the general molecular mechanism underlying the recurrence of CRC by using a CRC animal model harboring an *apc* mutation, a gatekeeper of CRC. Subsequently, the clinical uses of these mechanisms were tested by using tumor organoids derived from CRC patients harboring differential and diverse mutations [5]. Tumor organoids derived from mouse or patient tumor cells are a powerful model for cancer studies [79,80].

Competition among various cancer cell clones results in the development of cancer. Surviving clones during anti-cancer treatment can be expanded, which results in cancer recurrence. 

## 9. Co-Culture System of Organoids

Cancer immunotherapies targeting immune checkpoints, such as CTLA4 and the PD-1/PD-L1 axis, have presented substantial clinical benefits for various cancer types, such as melanoma, leukemia, and lung cancer [81,82]. In addition, recent studies have shown that cell therapies, such as CAR-T and –NK-cell therapies, are a promising therapeutic strategy for cancer treatment with good clinical outcomes [83,84,85,86]. However, by lacking in vitro model systems, the present research was restricted and difficulties in the development of cancer immunotherapy models were faced by the researchers. Recently, many researchers have focused on the establishment of co-culture systems using 3D-tumor organoids with immune cells [87,88,89]. Dijkstra et al. (2018) generated the co-culture of tumor-reactive T cells with tumor organoids. Their co-culture system showed an unbiased platform for tumor-reactive T cells and provided a means by which to access the sensitivity of tumor cells to a T-cell-mediated attack at the level of the individual patient, shown by how T cells can efficiently assess and kill the corresponding tumor organoids. Moreover, the protocols used for tumor organoid-T-cell co-culture systems were reported by Cattaneo et al. (2020). Tumor organoids derived from NSCLC patients and microsatellite-instable CRCs grown with CD8^+^ T cells can be easily grown [90]. Therefore, the research has enabled the establishment of ex vivo test systems for T-cell-based immunotherapy at the level of the individual patient

## 10. Conclusions

The recent accessibility of innovative genomic analysis technologies, including single-cell RNA sequencing, has redefined our understanding of biological heterogeneity across cancer subtypes. Intra-/inter-tumoral heterogeneity that results from somatic mutations and differential protein expressions and modifications occurred in the process of tumorigenesis [91]. Therefore, the existence of new cancer model systems representing the heterogeneity of cancer cells are necessary to enhance our understanding of cancer biology and develop effective anti-cancer agents. Recently, intensive, novel approaches have been used to establish advanced in vitro and in vivo experimental applications representing the heterogeneity of cancer cells, including 3D tumor organoids derived from tumor cells in genetic mouse models or patient-specific tumors and PDTX mouse models. The innovative model systems show advanced rather than conventional applications and present their own advantages; every model system exhibits intrinsic limitations. Therefore, the selection of appropriate model systems suitable for each study is fundamentally important to each study’s success.

## Data Availability

Not applicable.
